# Know the enemy and know yourself: Addressing cryptic fungal pathogens of humans and beyond

**DOI:** 10.1371/journal.ppat.1011704

**Published:** 2023-10-19

**Authors:** Jacob L. Steenwyk, Antonis Rokas, Gustavo H. Goldman

**Affiliations:** 1 Howards Hughes Medical Institute and the Department of Molecular and Cell Biology, University of California, Berkeley, Berkeley, California, United States of America; 2 Department of Biological Sciences and Evolutionary Studies Initiative, Vanderbilt University, Nashville, Tennessee, United States of America; 3 Faculdade de Ciencias Farmacêuticas de Ribeirão Preto, Universidade de São Paulo, São Paulo, Brazil; Vallabhbhai Patel Chest Institute, INDIA

*“If you know the enemy and know yourself*, *you need not fear the result of a hundred battles*. *If you know yourself but not the enemy*, *for every victory gained you will also suffer a defeat*. *If you know neither the enemy nor yourself*, *you will succumb in every battle*.*”*—*Sun Tzu*, *The Art of War*

Fungal pathogens threaten human welfare, including human and animal health and global food security. For example, ameliorating crop loss due to fungal plant pathogens would feed nearly 600 million more people [1]. Fungal infections of humans are difficult to diagnose and treat, potentially leading to serious illness and death, especially in immunocompromised individuals; case in point, there are an estimated 1.7 million deaths per year and more than 150 million severe fungal infections worldwide [2]. These issues may be exacerbated due to climate change. Reflecting the importance of combatting and preventing infection diseases caused by fungi, the World Health Organization recently released a list of priority fungal pathogens (https://www.who.int/publications/i/item/9789240060241). Accordingly, fungi that cause disease have become a major research focus, resulting in a deeper, but still incomplete, understanding of virulence and the cadre of pathogenic species.

Most human fungal pathogens are opportunistic and affect immunocompromised individuals. This suggests that any fungus capable of growing in the human body and overcoming or evading a compromised immune system could cause disease. Supporting this notion, advances in DNA sequencing of clinical isolates have led to the realization that cryptic fungal species—morphologically indistinguishable from known pathogens but genetically distinct—are an underappreciated source of infections [3–6]. Cryptic species have been identified in several fungal genera of agricultural and biomedical significance, such as plant pathogens (e.g., *Fusarium*) [7] and human pathogens (e.g., *Aspergillus*; Table 1) [3]. Due to morphological similarities (Fig 1), cryptic fungi are difficult to diagnose using typical microbiology techniques in the clinical setting, resulting in an incomplete understanding of their epidemiology and clinical burden.

**Fig 1:**
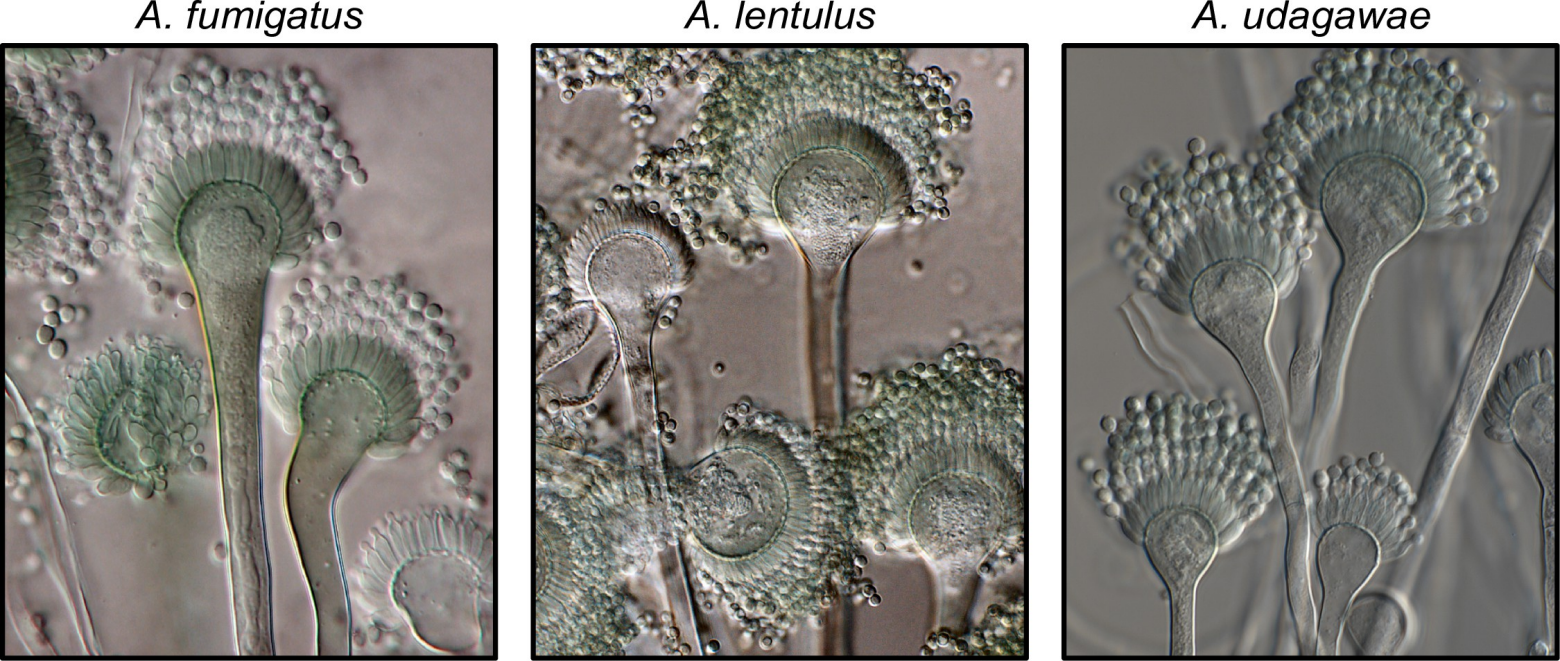
Exemplary known and cryptic pathogenic species of *Aspergillus*. (Left) *Aspergillus fumigatus* is a well-known and major human fungal pathogen. The cryptic species (Middle) *Aspergillus lentulus* and (Right) *Aspergillus udagawae* can also cause human disease but may be difficult to diagnose owing to their morphological similarity to *A*. *fumigatus*. Images were kindly provided by Dr. Jos Houbraken.

**Table 1:** Exemplary cryptic *Aspergillus* species that have been associated with human disease.

Section	Cryptic species	Morphologic identification	Underlying patient condition	Reference
*Aspergillus*	*A*. *montevidensis*	*Aspergillus* sp.	Heart transplantation	[3]
*Circumdati*	*A*. *persii*	*Aspergillus* sp.	Unknown	[5]
	*A*. *tanneri*	*Aspergillus* sp.	Chronic granulomatous disease	[20]
	*A*. *westerdijkia*	*Aspergillus* sp.	Unknown	[5]
*Flavi*	*A*. *alliaceus*	*Aspergillus* sp.	Non-Hodgkin lymphoma	[3,5]
	*A*. *nomius*	*Aspergillus* sp.	Waldenstrom’s disease	[3]
	*A*. *pseudocaelatus*	*Aspergillus* sp.	Nasopharyngeal carcinoma, postradiotherapy	[6]
	*A*. *tamarii*	*Aspergillus* sp.	Left ear cholesteatoma with attic perforation	[6]
*Fumigati*	*A*. *arcoverdensis*	*A*. *fumigatus*	HIV	[3]
	*A*. *ellipticus*	*A*. *fumigatus*	Pulmonary cavity	[3]
	*A*. *fumigatiaffinis*	*Aspergillus* sp.	Unknown	[5]
	*A*. *lentulus*	*A*. *fumigatus*	Cirrhosis; thymoma	[3]
	*A*. *novofumigatus*	*Aspergillus* sp.	Unknown	[5]
	*A*. *udagawae*	*A*. *fumigatus*	Unknown	[5]
	*A*. *viridinutans*	*A*. *fumigatus*	Unknown	[5]
*Nidulantes*	*A*. *amoenus*	*Aspergillus* sp.	Chronic obstructive pulmonary disease	[6]
	*A*. *austroafricanus*	*Aspergillus* sp.	Nil	[6]
	*A*. *latus*	*A*. *nidulans*, *A*. *spinulosporus*, and A. quadrilineatus*	Chronic obstructive pulmonary disease; chronic granulomatous disease; allgeric bronchopulmonary aspergillosis; asthma	[4,9]
	*A*. *sydowii*	*Aspergillus* sp.	Gastroesophageal reflux disease, steroid-dependent asthma; bronchiectasis, chronic obstructive pulmonary disease, ischemic heart disease	[5,6]
	*A*. *tabacinus*	*Aspergillus* sp.	Nil	[6]
*Nigri*	*A*. *awamori*	*Aspergillus* sp.	Unknown	[5]
	*A*. *brunneoviolaceus*	*Aspergillus* sp.	Nil	[6]
	*A*. *costaricensis*	*Aspergillus* sp.	Hyperthyroidism, multiple thyroid nodules	[6]
	*A*. *luchuensis*	*Aspergillus* sp.	Unknown	[5]
	*A*. *tubingensis*	*Aspergillus* sp.	HIV	[3]
	*A*. *welwitschiae*	*Aspergillus* sp.	Nasopharyngeal carcinoma; Alzheimer’s disease, old pulmonary tuberculosis, bronchiectasis	[6]
*Restricti*	*A*. *restrictus*	*Aspergillus* sp.	Lichen planus, psoriatic arthritis	[6]
*Terrei*	*A*. *alabamensis*	*Aspergillus* sp.	Unknown	[5]
	*A*. *carneu*	*Aspergillus* sp.	Unknown	[5]
*Usti*	*A*. *calidoustus*	*Aspergillus* sp.	Unknown	[5]
	*A*. *insuetus*	*Aspergillus* sp.	Unknown	[5]
	*A*. *keveii*	*Aspergillus* sp.	Unknown	[5]

Species are listed alphabetically by section and then by species name. Most reports do not include the pathogen they were misidentified as or simply report “*Aspergillus* sp.,” underscoring the lack of specificity using morphology-based identification.

^*^The misidentification of *A*. *latus* isolates was inferred using MALDI-TOF, not morphology.

Here, major research avenues that aim to enrich our understanding of cryptic fungal pathogens—as well as other major pathogens—and diminish their impact on human welfare are discussed. Cryptic fungal pathogens from the genus *Aspergillus* are used as an example. We argue that the impact of fungal infections, especially among cryptic species for which little is known, can be reduced if we have an in-depth understanding of pathogens, humans, and the interaction between the two.

## 1) Know the enemy: Accurate and rapid diagnosis of fungal pathogens

Accurate diagnosis is a key first step to mitigating the burden of fungal pathogens. Traditional diagnosis methods use microscopy, histopathology, culturing, and MALDI-TOF [8]. Although helpful, these methods often lack specificity, especially for cryptic pathogens. Molecular typing—such as phylogenetic analysis of one or a few loci—has proven more accurate and brought cryptic pathogens to light [3,6]. However, few loci can, at times, lack insufficient information for accurate species determination. For example, *Aspergillus latus—*a cryptic pathogen and allodiploid hybrid that originated from a fusion event between *A*. *spinulosporus* and a close relative of *A*. *quadrilineatus*, two species that are closely related to *A*. *nidulans—*has gone largely undetected in the clinical setting due to the shortcomings of a single-locus typing in a hybrid genome [4]. Population genome sequencing and extensive phenotyping has revealed diagnostics for robust identification of *A*. *latus*, such as a large genome size, which can be detected using genome sequencing, fluorescence-activated cell sorting, or measuring conidia sizes [9].

Other genome-scale approaches can overcome the insufficient information in molecular typing of one or a few loci. For example, data-rich phylogenomics has revealed instances of misidentified human and plant pathogens in the genus *Aspergillus* [10]. The greater specificity of phylogenomics comes with the drawbacks of requiring more expertise (i.e., knowledge of processing and analysis of genomic data), resources (e.g., sequencing and computational resources), and time. In the clinical setting, rapid and cost-effective diagnosis is critical for lowering mortality rates [8]. An unexplored genome-scale alternative approach ripe for application to fungi is average nucleotide identity. This and similar alternative approaches could be coupled to (semi)automated genome sequencing, assembly, and analysis infrastructure, powered by robotics and cloud computing. Comparative analysis of diverse methods for diagnosing fungal infections will elucidate the most efficacious method, or combination thereof, for faithful diagnostics.

Beyond identification, examining phenotypic profiles among pathogens will elucidate other key aspects of pathogen biology relevant to combatting and preventing disease. This is particularly true for cryptic fungal pathogens whose phenotypic profiles remain largely unknown, and numerous examples have revealed cryptic fungal pathogens have distinct drug resistance profiles compared to close relatives [4–6]. For example, the cryptic pathogen *A*. *latus* is substantially more drug resistant to caspofungin than reference isolates of *A*. *nidulans* [9]. Considering strain heterogeneity—phenotypic variation within a species, including variation in virulence and resistance to antifungal drugs—has emerged as an important consideration when studying microbial pathogenicity [4,9].

## 2) Know yourself: Understanding the human influence and patient populations

Although most efforts aim to prevent and treat fungal diseases, humans can sometimes unwittingly contribute to their spread. For example, hospital-associated outbreaks of invasive fungal infections have been linked to factors such as hospital construction, indoor water damage, inadequate air filtration, and transmission via contaminated surfaces [11]. Other factors may impact fungal diseases, such as human-to-human transmission, the influence of diet on modulating the abundance of certain fungi in the mycobiome, and the rise of antifungal resistance mainly through the abundant usage of fungicides with a similar mechanism of action in agriculture, confounded by the ability of some fungi to evolve drug resistance rapidly [12,13]. For example, genome sequencing efforts have revealed that drug-resistant environmental isolates of *A*. *fumigatus*—wherein resistance was likely acquired from agricultural use of first-line clinical azoles—can cause human disease [13].

Consideration of the patient population and genetic backgrounds is also crucial for understanding the role of host biology in disease susceptibility [3,5]. For example, patients with chronic granulomatous disease are at greater risk of *A*. *nidulans* infections [4,9]. Of note, the association between cryptic pathogens and certain patient populations remains largely unknown due to the absence of highly specific detection methods in the clinical setting, as discussed in the previous section.

## 3) Know the enemy and know yourself: Elucidating the immune response to pathogens will inform disease management strategies

Investigating the interaction between fungal pathogens and the host immune system offers valuable insights into mechanisms of pathogen clearance or persistence. Studies tackling this issue have revealed key immunological checkpoints necessary for pathogen recognition, such as a sufficient number of properly functioning myeloid phagocytes, conidial engulfment by alveolar macrophages, and proper activation of pro-inflammatory cascades via detection of β-1,3-glucan, a sugar found on the germinating conidia, through a dectin 1–dependent recognition process [14]. Considering the site of infection, as immune defenses differ between infections occurring at the skin or mucous membranes versus deeper anatomical sites, will also be important [14].

Immune cells can also launch direct assaults on microbes. Examination of NETosis, wherein neutrophils release extracellular traps to kill microbes, revealed substantial heterogeneity among species and strains of *Aspergillus* species from the section *Nidulantes*; for example, among the strains tested, NETosis of the cryptic pathogen *A*. *latus* resulted in more pathogen lysis than NETosis of *A*. *spinulosporus* [4]. Certain inborn errors in immune system function render some populations more susceptible than others and should be considered in these studies. For example, those with chronic granulomatous disease are susceptible to aspergillosis because immune cells have a reduced capacity to generate reactive oxygen species, a key component of the immune defense system [14]. Elucidating the interaction between host and pathogen will help pave the way for developing more targeted treatment and prevention regimes.

## 4) Beyond the bipartite of host and pathogen: The influence of the microbiome on health and disease

Investigating the interaction between fungal pathogens and the biology of the human host offer valuable insights into the clearance or persistence of pathogens within the human body [4]. However, fungal pathogens are just one of the many organisms in the human microbial ecosystem known as the microbiome. Exploring the microbiome, including the mycobiome, holds immense potential in elucidating the impact of diverse species involved in complex species–species interactions that can contribute to or prevent disease. Perturbations in the mycobiome, induced by antifungal treatments, can exacerbate allergic airway disease, indicating mycobiome imbalances contribute to disease severity [15]. Pathogens can also influence the mycobiome; for example, *A*. *fumigatus* colonization of the lung shifts microbiome composition to be more beneficial for fungal growth [16]. This and other findings suggest that targeting the mycobiome may have therapeutic potential.

Although most mycobiome studies have provided insights at the genus level, delving into species- and strain-level variations will offer a detailed understanding of how specific species and individual isolates thereof influence the mycobiome and human health. This issue is also prevalent in some traditional methodologies. For example, cryptic *Aspergillus* pathogens often go undetected because only genus-level identification is possible (Table 1). Accurate species and strain-specific identification is particularly important due to genomic and phenotypic strain heterogeneity among clinically relevant traits of pathogenic microbes [4,9,16]. As a result, some dimensions of the microbiome go undetected, limiting our understanding of the relationship between the microbiome and the host. These investigations will also shed light on the prevalence of cryptic fungal pathogens among healthy individuals, for which little is known. The occurrence of superinfections—coinfections of multiple pathogens, such as *Aspergillus* and SARS-CoV-2, which causes COVID-19—is becoming better appreciated and is associated with devastating mortality [17], underscoring the importance of considering complex species-species interactions.

## 5) The evolution of pathogenesis and identifying potential future threats

While the present article has predominantly addressed pathogens, especially cryptic ones, within the clinical setting, investigating nonpathogenic species remains essential [18]. This is due to the potential emergence of previously unrecognized species as disease-causing agents in clinical contexts. An illustrative example is *A*. *lentulus*, an opportunistic human pathogen with high mortality rates first described in 2005 [19]. Therefore, studying pathogenic species and closely related non-pathogens can provide valuable insights into the evolutionary dynamics underlying the emergence of pathogenicity [18].

Moreover, a broader understanding of the fungal lifestyle may provide insight into ecologies and life histories that tend to give rise to pathogens. For example, growth in warming climates may predispose certain fungi to better grow at the human body temperature. Thus, providing a geographic context to studies of microbial pathogens and their close relatives will not only elucidate epidemiologic patterns among known pathogens, but also potentially help identify species that pose a future threat to human health. Ecologies are largely unknown among cryptic pathogens, further obfuscating our understanding of their habitat and our capacity to predict outbreaks. Furthermore, comprehensive exploration of diverse fungi can aid in identifying species that may pose a future threat to human welfare due to possessing pathogen-like characteristics, such as growth at human body temperature and rapid evolution of drug resistance.

## Concluding remarks: Embracing the complexity of the fungal pathogen problem

Addressing the aforementioned challenges relies on overcoming the major challenge of establishing the necessary infrastructure for accurate diagnostic testing and dissemination of findings. Such infrastructure will facilitate monitoring disease burden, elucidating pathogen epidemiology, identifying susceptible patient populations, illuminating geographic locales with increased prevalence of certain pathogens, and facilitating the identification of early outbreaks and emerging pathogens [12]. In the context of cryptic pathogens, basic knowledge gaps, such as clinical frequency, will come to light and help inform fungal pathogen priority lists like the one established by the World Health Organization. More broadly, accurately identifying microbial pathogens is the key first step in treating infection.

Insights from other surveillance systems can guide tackling this Herculean objective. For example, a platform like Nextstrain (https://nextstrain.org) or Microreact (https://microreact.org/)—web-based applications with geographic, temporal, and genomic information for comparative and phylogenetic analysis of influenza, Dengue, SARS-CoV-2, and other pathogens—can serve as inspiration for a fungal-focused platform. More broadly, integrating other dimensions of data, such as host genetics and electronic health records as well as microbiome and pathogen data, will offer a comprehensive data set for researchers teams across the globe to address the fungal pathogen problem.
